# Expedited Development Programs at the Food and Drug Administration: Insights and Opportunities

**DOI:** 10.1007/s43441-021-00258-z

**Published:** 2021-02-02

**Authors:** Grace Collins, Mark Stewart, Ellen Sigal, Jeff Allen

**Affiliations:** 1 Friends of Cancer Research, Washington, DC USA; 2 1800 M Street NW, Suite 1050 South, Washington, DC 20036 USA

Timely access to therapies that treat serious illnesses is critical for patients, particularly for those with rare or serious disease types that have no current treatments. Congress and the Food and Drug Administration (FDA) have addressed this by periodically establishing programs designed to expedite different steps associated with the development and review of drugs and biologics. Over time, these efforts have been effective in getting new treatments to patients faster than traditional approval processes [1]. Here, we present an analysis of original therapeutic oncology agents approved between January 1, 2013, and September 4, 2020, to understand how expedited programs are utilized in oncology, a disease area where these pathways have been utilized the most.

**Fig. 1:**
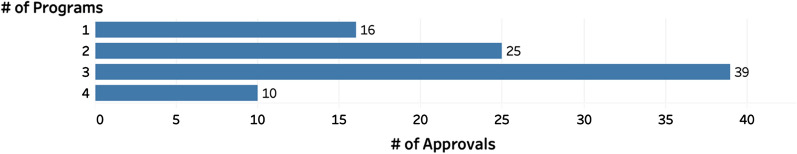
Utilization of expedited programs alone or in combination for oncology drugs. Expedited programs are rarely used in isolation and are often combined with one or more other expedited programs

We analyzed development timelines using publicly available FDA review documents through the online database Drugs@FDA. We compiled Investigational New Drug (IND) Application submission dates, New Drug Application (NDA) and Biologics Licensing Application (BLA) receipt dates, approval dates, and noted use of Fast Track, Breakthrough Therapy Designation, Priority Review, and Accelerated Approval. During this 7.5-year period, the FDA approved 98 original oncology treatments [2]. To gain a comprehensive view of how expedited programs are utilized, we examined a time period during which all pathways were active, beginning in 2013 when the most recently established pathway, Breakthrough Therapy Designation, became available for use. We note that the number of oncology drug approvals over the past 4 years (2017–2020 | *n* = 61) increased 65% compared to the 4 years before that (2013–2016 | *n* = 37). The two most used pathways were Priority Review (86%, *n* = 84) and Breakthrough Therapy Designation (54%, *n* = 52), and 92% (*n* = 90) of all approvals used at least one expedited pathway (Table 1). Our analysis shows expedited pathways were rarely used alone. 76% of expedited approvals were approved using a combination of two or more expedited pathways (Fig. 1).

**Table 1:** Utilization of expedited programs by year for oncology drug approvals

	Year of approval (*n* = # of approvals)							
	2013 (*n* = 8)	2014 (*n* = 8)	2015 (*n* = 16)	2016 (*n* = 5)	2017 (*n* = 16)	2018 (*n* = 17)	2019 (*n* = 10)	2020 (*n* = 18)
Fast Track	7 (87.5%)	4 (50.0%)	7 (43.8%)	2 (40.0%)	7 (43.8%)	7 (41.2%)	3 (30.0%)	6 (33.3%)
Breakthrough Therapy Designation	2 (25.0%)	5 (62.5%)	5 (31.3%)	5 (100.0%)	12 (75.0%)	6 (35.3%)	6 (60.0%)	11 (61.1%)
Priority Review	6 (75.0%)	8 (100.0%)	12 (75.0%)	5 (100.0%)	14 (87.5%)	15 (88.2%)	9 (90.0%)	15 (83.3%)
Accelerated Approval	2 (25.0%)	7 (87.5%)	5 (31.3%)	4 (80.0%)	6 (37.5%)	3 (17.6%)	7 (70.0%)	10 (55.6%)

Percentages total greater than 100% because multiple expedited programs can be used for a single drug. Expedited pathways were established overtime to reflect the evolving and modernizing landscape of regulatory science. Priority Review and Accelerated Approval were established in 1992, Fast Track in 1997, and, most recently, Breakthrough Therapy Designation in 2012

**Fig. 2:**
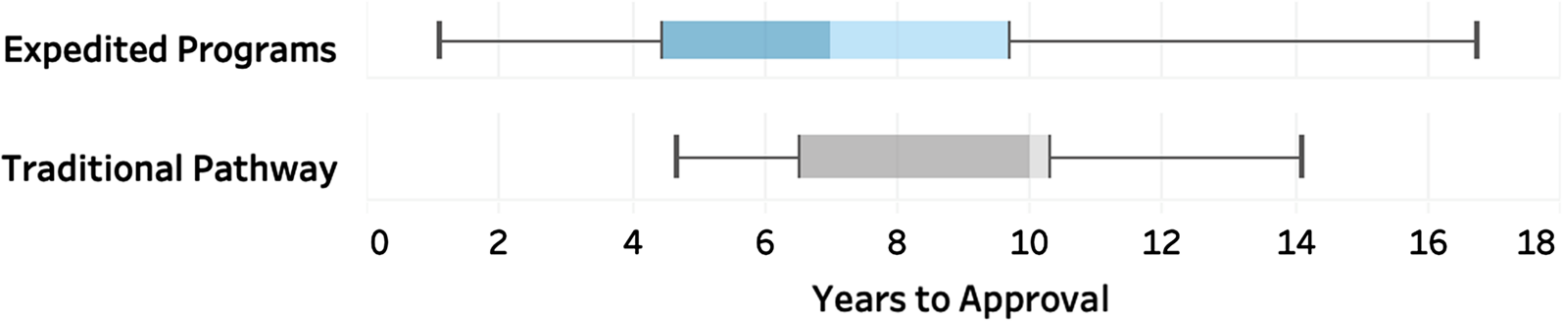
Median years to approval for oncology drugs utilizing expedited programs versus the traditional approval pathway. Use of expedited programs shortened median time to approval for qualifying drugs (Expedited Development = 6.58 years) compared to drugs that do not qualify for an expedited program (Traditional Pathway = 10 years)

When comparing the median development time (IND submission to NDA/BLA submission) of novel agents using expedited programs (2013–2020 | *n* = 90) to novel approvals that used traditional approaches (2013–2020 | *n* = 8), we found the use of expedited programs reduced the median development time by 3.4 years and shortened median review time by 4 months (Fig. 2). For approvals that used only one expedited program (*n* = 16), the median time to development was 9.62 years, compared to 5.76 years for those approved using two or more expedited approaches (*n* = 74).

By evaluating the processes associated with these programs based on the wealth of experience gained over the past decade in oncology, insights into their effectiveness and opportunities for improvement emerge. The shifting utilization and utility of these pathways must be considered in the context of current scientific capabilities and cutting-edge drug development procedures. While Priority Review has consistently been used since 2013, the percent of approvals using Fast Track significantly decreased overtime. More than half of expedited approvals in our analysis used a combination of expedited programs as opposed to one alone. For example, of the 52 drugs that used Breakthrough Therapy Designation and Priority Review, 19 used Fast Track, and 34 used Accelerated Approval. While there are overlapping benefits when using these programs in combination, there are also duplicative application and administrative processes for those with similar requirements. Despite these redundancies, sponsors continue to use programs in combination. To ensure optimal use of all programs—alone or in combination—FDA’s resources must be allocated most efficiently and developers’ processes optimized. To that end, it may be worth creating a more streamlined process to avoid redundancy in the administrative processes associated with pathways frequently used together. A more streamlined process would maximize resources and time for both FDA and Sponsors to continue driving innovation and prioritize development of promising drugs.

In summary, expedited mechanisms effectively facilitate development and review processes and shorten time to approval for original therapeutic approvals in oncology. They collectively help provide effective new treatments to patients faster than the traditional approval processes. Many of these new therapies have since demonstrated long term population-level benefits, such as a significant reduction in overall lung cancer mortality [3]. A delay for these therapies would result in a lag in such benefits for potentially thousands of patients. The frequent use of expedited programs in oncology provides a wealth of experience and learning which can be applied to optimize the use of expedited programs in other serious disease spaces where there are few or no available treatments.

To ensure the benefit of these pathways evolves to reflect current science and technological capabilities, it is essential that FDA has adequate resources to optimize these expedited programs. As steps for the seventh reauthorization of the Prescription Drug User Fee Act begin, there is an opportunity to modernize approaches to reflect the current state of drug development and regulation. A periodic review of these programs will allow planning for future capacity and ensure processes associated with their use are optimized. In an era where we will likely see an expansion of emerging new therapies [4, 5] aimed at treating serious and life-threatening diseases, it is critical to ensure expedited programs continue to facilitate the science, provide appropriate access for patients, and are sustainable.
